# Supplementary material for: MetaRibo-Seq measures translation in microbiomes
Source: Nat Commun. 2020 Jun 29;11:3268. doi: 10.1038/s41467-020-17081-z (PMC7324362; doi:10.1038/s41467-020-17081-z)
Supplement: Supplementary file 10 — Supplementary Data 7 [file 41467_2020_17081_MOESM10_ESM.zip › File2/Confidence_VeryHigh_Taxonomy/165206_out.krona.html]

Javascript must be enabled to view this page.

members
magnitude
magnitudeUnassigned
count
unassigned
taxon
rank

165206\_out

7

7
superkingdom
2

phylum
1239
7

3

SRS020233\_contig\_number\_28212SRS075984\_contig\_number\_21954SRS142542\_contig\_number\_11863
species
1263007

4
class
186801

4
order
186802

2
186803
family

species
1898203
2

SRS045004\_contig\_number\_contig-100\_2413.194842SRS893378\_contig\_number\_1391

1
family
186806

1730
genus
1

species
165185
1

SRS057478\_contig\_number\_1678

1
family
541000

genus
1263
1

2293146
species

SRS050925\_contig\_number\_32391
1
